# Kinetic and toxicological effects of synthesized palladium(II) complex on snake venom *(Bungarus sindanus*) acetylcholinesterase

**DOI:** 10.1590/1678-9199-JVATITD-2020-0047

**Published:** 2021-04-09

**Authors:** Mushtaq Ahmed, Shahan Zeb Khan, Naila Sher, Zia Ur Rehman, Nadia Mushtaq, Rahmat Ali Khan

**Affiliations:** 1 Department of Biotechnology, University of Science and Technology Bannu-KPK, Pakistan; 2 Department of Chemistry, Quaid-i-Azam University, Islamabad, Pakistan; 3 Department of Botany, University of Science and Technology Bannu-KPK, Pakistan

**Keywords:** Palladium (II) complex, Snake venom, Acetylcholinesterase, Inhibition, Kinetics

## Abstract

**Background:**

The venom of the krait (*Bungarus sindanus*), an Elapidae snake, is highly toxic to humans and contains a great amount of acetylcholinesterase (AChE). The enzyme AChE provokes the hydrolysis of substrate acetylcholine (ACh) in the nervous system and terminates nerve impulse.

Different inhibitors inactivate AChE and lead to ACh accumulation and disrupted neurotransmission.

**Methods:**

The present study was designed to evaluate the effect of palladium(II) complex as antivenom against krait venom AChE using kinetics methods.

**Results:**

Statistical analysis showed that krait venom AChE inhibition decreases with the increase of Pd(II) complex (0.025-0.05 µM) and exerted 61% inhibition against the AChE at a fixed concentration (0.5 mM) of ACh. Kinetic analysis using the Lineweaver Burk plot showed that Pd(II) caused a competitive inhibition. The compound Pd(II) complex binds at the active site of the enzyme. It was observed that *K*_*m*_ (Michaelis-Menten constant of AChE-ACh into AChE and product) increased from 0.108 to 0.310 mM (45.74 to 318.35%) and *V*_*max*_ remained constant with an increase of Pd(II)

complex concentrations. In AChE *K*_*Iapp*_ was found to increase from 0.0912 to 0.025 µM (29.82-72.58%) and did not affect the *V*_*maxapp*_ with an increase of ACh from (0.05-1 mM). *K*_i_ (inhibitory constant) was estimated to be 0.029* *µM for snake venom; while the *K*_*m*_ was estimated to be 0.4 mM. The calculated IC_50_ for Pd(II) complex was found to be 0.043 µM at constant ACh concentration (0.5 mM).

**Conclusions:**

The results show that the Pd(II) complex can be deliberated as an inhibitor of AChE.

## Background

Acetylcholinesterase (AChE) (EC.3.1.1.7) is a serine hydrolase that is found in peripheral and central tissues; sensory and motor fibers; cholinergic and non-cholinergic fibers; and in nerves and muscles. In motor neurons, AChE presents higher activity as compared to sensory neurons [1]. Its main biological role is the hydrolysis of ACh into acetate and choline to terminate impulse transmission. AChE has a remarkably high specific catalytic activity, especially for a serine hydrolase, each molecule of AChE degrades about 25000 molecules of ACh per second [2,3]. AChE consists of two sites i.e. the peripheral site (p-site) and the anionic binding site [4]. The anionic binding site has two further domains: the ionic site that consists of serine and histidine, and the esteratic site that contains glutamate [4,5]. The serine residue of the ionic site is responsible for substrate hydrolysis while histidine residue acts as base and acid during hydrolytic processes. In the esteratic site, the glutamate residue clutches the cationic head of the substrate ACh [4,5]. The acetylcholine (ACh) is a neurotransmitter that is found in many autonomically innervated organs, in the neuromuscular junction, in many synapses, in all autonomic ganglia, and in the central nervous system. The neurotransmitter ACh plays a major role in physiological events and is inactivated by AChE through enzymatic breakdown [6,7]. ACh is degraded into choline and acetate by AChE [8]. Alzheimer's disease (AD) patients can be treated by degeneration of this pathway [9].

Neurodegenerative diseases induce changes in the central nervous system (CNS) [10]. As it is widely known, AD is a neurodegenerative disorder that causes dementia among elderly people [11,12]. Acetylcholinesterase inhibitors (AChEI) can help in the treatment of numerous pathologies, including glaucoma, Lewy body dementia, myasthenia gravis, and AD [13]. AChE inhibition is known to treat AD by improving functions and the ACh amount in the cholinergic synapses. 

Snake venom AChE is monomeric while in all mammals it is a multimeric form, with two or more subunits [14,15]. Snakes of the Elapidae family have a large number of ACHE enzymes [16] and are found in several parts of the world [17].*Bungarus* *sindus*venom contains about 747,000 Ellman’s units per g of dry venom of AChE-like activity, and is one of the richest venoms with such activity [16]. Therefore, for analyzing AChE mechanism,*Bungarus* *sindus*venom is an excellent model [18].

According to the World Health Organization, 5.4 million snake bites are reported each year globally. Out of the 2.5 million people affected, about 125,000 of them die. In rural areas, death from snakebite is a significant public health problem and people of these areas have no access to antivenom [19]. There are approximately 10,000 to 50,000 snakebite-related deaths in India every year [20]. In Asia, most of the deaths attributable to snakebite involve members of the family Elapidae [20,21]. To treat snakebite victims, antivenom is the most specific therapy [22]. Some bio-organometallic showed how rational ligand can be designed for new improved therapies [23].

Heteroleptic Pd(II) complex compounds were the first molecules that were discovered for therapeutic purposes [24]. Recently, the literature has shown that these compounds possess neuroprotective effects, and thus increase the scientific interest in the pharmaceutical industry [25,26]. A variety of compounds from different origins were discovered and reported to be effective against several diseases, including the neurological ones, such as AD [27,28].*In vivo*and*in vitro*studies have proven the medicinal value of these compounds [29].

The heterocyclic organic compounds bearing coumarin possess an important biological role in medicinal chemistry [30]. They are commonly used as cosmetics, food additives, perfumes, pharmaceuticals, laser dyes, and optical brighteners [31,32]. Recent studies have shown that Pd(II) complexes are about 105 times more combative as compared to Pt (II) analogs; the lower anti-AChE activity of Pd compounds has been ascribed to hydrolysis of the leaving groups that separate readily in solution, and leading to reactive species far from their pharmacological targets [33&093;. Due to the substantial similarity in the coordination chemistry of palladium [Pd(II)] and platinum [Pt(II)] the development of Pd(II)-based drugs has received much importance after the cisplatin success in the cancer therapy [34]. The carbonyl group in Pd(II) complex acts as antiglycation,. Almost all Pd(II) complexes showed antiglycation potential and provoked suppression of disorders [34]. From the pharmacological point, Pd(II) complexes may be considered better inhibitors than those free of Pd(II), which are based on the potential ability of ligands to reduce the toxicity of free metal ion [34]. Pd(II) complex compounds are a class of irreversible AChE enzyme inhibitors that form a covalent bond between the esteratic site and phosphoryl group of the enzyme [35]. Phosphorus compounds irreversible bind to AChE by forming a covalent bond between the phosphoryl group and the esteratic site of the enzyme [36]. The present study aimed to investigate the antivenom potential of Pd(II) complex compound on krait (*Bungarus sindanus*) venom acetylcholinesterase (AChE) by *in vitro* methods.

## Methods

### Materials

AChE, butyrylthiocholine iodide, DTNB [5,5´-dithiobis(2-nitro-benzoic acid)], sodium dihydrogen phosphate, disodium hydrogen phosphate bovine serum albumin, Tris (hydroxymethyl aminomethane) and Coomassie Brilliant blue R-250 were purchased from Sigma (USA). All other reagents used were of analytical grade.

### Synthesis of palladium(II) complex

The Pd(II) complex was synthesized as previously reported [37]. A stoichiometric amount of palladium(II) chloride reacted with diphenyl-*p*-tolyl phosphine and 4-diphenylmethylpiperazine-1-carbodithioate using methanol and acetone as solvents. The synthesized Pd(II) compound was confirmed by using various technique i.e. CHNS, FT-IR and multinuclear NMR analysis as reported in our previous paper [37]. 

### Preparation of stock solution 

To prepare stock solution 2.5 mM Pd(II) complex was dissolved in 2 mL DMSO which was further diluted to several solutions of different concentrations 0.025, 0.037, and 0.05 (µM).

### Venom 

Male and female mature live snakes (*Bungarus sindanus*) were obtained from Thatta District of Sindh Province, Pakistan. A small amount of snake venom was milked manually, mixed, immediately lyophilized, powdered and then mixed with 1 mL dH_2_O and kept at −20°C for future activities. The study was approved by the Departmental Ethical Approval Committee. 

### Protein determination 

Bovine serum albumin was used as standard. The protein was assayed by following the procedure of Bradford [38]. About 2 µg protein was used for the enzymatic assay.

### Acetylcholinesterase assay 

In the present study, the anti-AChE activities of the Pd(II) complex were confirmed by the standard methodology of Ahmed [39], which was modified by Rocha [40]. AChE hydrolysis rates (V) were estimated at different substrate ACh concentrations (0.05-1 mM) in 1 mL assay mixture with 10 mM DTNB [5,5'-dithiobis-(2-nitrobenzoic acid)] and 50 mM phosphate buffer, pH 7.4 at 25°C. About 20 µL of diluted*Bungarus sindanus*venom was also added to the above mentioned reaction mixture. 

The mixtures were then incubated at 37^o^C for 5 minutes. After the addition of the substrate (ACh), the enzyme-substrate (AChE-ACh) reaction started immediately. The AChE-ACh hydrolysis rate was analyzed by the formation of thiolate di-anion of DTNB after every 15 seconds during 90 seconds using a double beam spectrophotometer UV-1602, BMS biotechnology medical service. The measurement of the amount of the yellow color which develops from the AChE-ACh reaction revealed the AChE activity.* *All samples were run in triplicate.

### Kinetic determination

The Pd(II) complex-AChE interaction rate was determined by using the Lineweaver [41] double reciprocal plot, by plotting 1/rate of enzyme activity (1/V) against 1/substrate (1/S) analyzed over a range of different substrate ACh concentrations (0.05-1 mM) in the absence and presence of Pd(II) complex (0.025, 0.037 and 0.05µM). Michaelis Menten constant (*K*_*m*_ ) was determined by two different plot of 1/V versus 1/S [41] and V versus V/S [42,43]. The values of inhibition constant (*K*_i_) were determined by using Cornish-Bowden plots of S/V versus [I]. IC_50_ was estimated at fixed ACh concentration (0.5 mM), by following Dixon [44] plot using 1/V versus [I].

### Statistical analysis 

Statistical analysis was performed using one-way ANOVA, which was followed by post-hoc analysis (Duncan multiple range tests). The difference was considered to be significant for p < 0.05.

## Results

### Pd(II) complex anti-acetylcholinesterase activity

**Figure 1.**
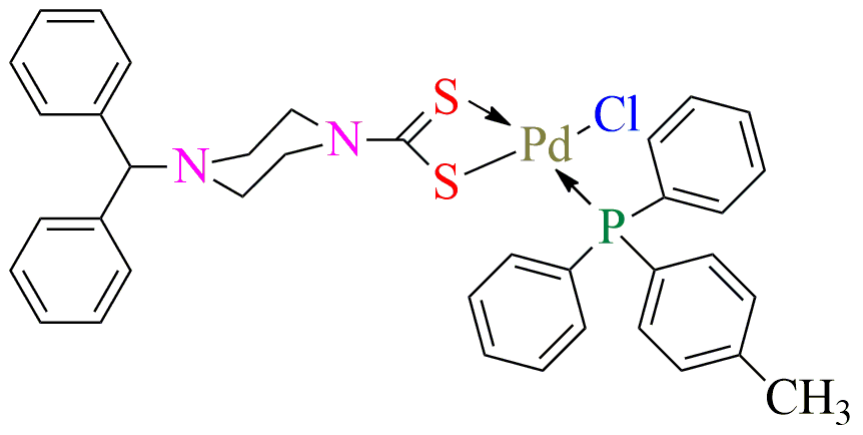
Chemical structure of Pd(II) complex.

**Figure 2.**
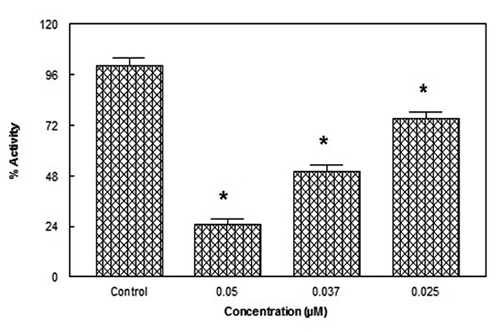
Concentration-dependent inhibition of krait snake venom AChE in the absence and presence of Pd(II) complex were measured at 412 nm by using 0.5 mM substrate in 1 mL assay solution with 50 mM phosphate buffer (pH 7.4). 10 mM DTNB [5,5-dithiobis(2-nitronenzoic acid)] was pre incubated for 10 min before 0.5 mM substrate addition. All experiments were repeated at least two times and similar results were obtained. *p < 0.05, significantly different from control.

In the present study Pd(II) complex (Figure 1) inhibited snake venom AChE. We observed that at a fixed concentration 0.5 mM of substrate, Pd(II) complex showed 61% inhibition of AChE activity (Figure 2).

### Determination of IC_50_

**Figure 3.**
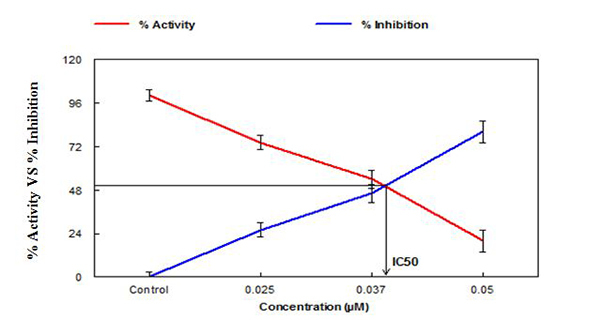
A plot of the percentage residual activity in the absence and presence of Pd(II) complex after 10 minute incubation at 37°C versus various concentration of Pd(II) complex. 0.5 mM AcSCh was used as a substrate for snake venom AChE. The results represent the mean of three different experiments done in triplicate.

In the present study, at a fixed concentration of 0.5 mM of the substrate (Figure 3), Pd(II) complex showed 50% inhibition. IC_50_ calculated by plotting the percentage of residual activity versus inhibitor concentration. The IC_50_ value for Pd(II) complex was found to be 0.043 µM (Table 1). 

**Table 1.** Comparative study of kinetic parameters of cholinesterase inhibition by Pd(II) complex. *V*_*max*_ and *V*_*max*_ /*K*_*m*_ and of snake venom AChE at fixed 0.5 mM substrate (ASCh).

Parameters	Snake venom AChE
*K*_i_ (µM)	0.029
IC_50_ (µM)	0.043
*K*_*m*_ (mM)	0.4
*V*_*max*_	16.6
*V*_*max*_ /*K*_*m*_	41.5

*K*_i_: inhibition constant; IC_50_ *;* half of maximal inhibitory concentration; *K*_*m*_: Michaelis-Menten constant.

### **Determination of *K***_*m*_

**Figure 4.**
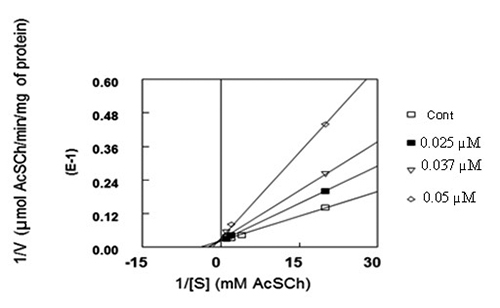
Pd(II) complex caused a competitive type of inhibition of krait snake venom AChE. Data are expressed in the form of Lineweaver-Burk (reciprocal of enzyme velocity versus reciprocal of AcSCh) plot. The results represent the mean of three different experiments done in triplicate by using different concentration of Pd(II) complex.

In the present study, *K*_*m*_ (Michaelis-Menten constant) was deliberate by using the Lineweaver-Burk plot and was estimated to be 0.4 mM (Figure 4).

### **Effects of Pd(II) complex on *K***_*m*_ **and *V***_*max*_

Kinetics analysis revealed that Pd(II) caused competitive type inhibition against AChE (Figure 4). The *K*_*m*_ increased from 0.108 to 0.310 mM (45.74 to 318.35%) and the *V*_*max*_ was unchanged (Table 2).

**Table 2.** Effect of Pd(II) complex on *K*_*m*_ and *V*_*max*_ of *Bungarus sindanus* (krait) venom AChE.

**Pd (II) complex *(*µM/mL)**	*K*_*m*_ (mM)	% Increase	*V*_*max*_ (µmol/min per mg protein)
0	0.0741	0	20
0.025	0.108	46	20
0.037	0.230	211	20
0.05	0.310	320	20.4

### **Effects of palladium(II) complex on *K***_*Iapp*_ **and *V***_*maxapp*_

**Figure 5.**
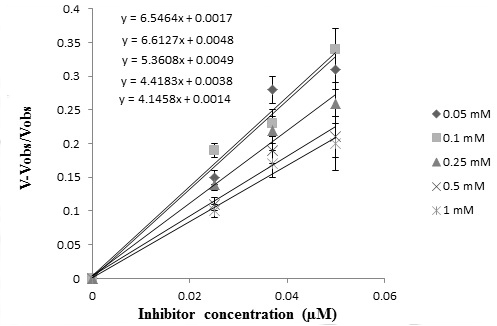
Effects of Pd(II) complex on *KIapp* and *Vmaxapp*. *KIapp* was found to increase from 0.0912 to 0.025 µM (29.82-72.58%) while *Vmaxapp* remained unchanged with increase of substrate concentration (0.05-1 mM).

In the present study, *K*_*Iapp*_ and *V*_*maxapp*_ were observed for AChE (Figure 5). *K*_*Iapp*_ was found to increase from 0.0912 to 0.025 µM (29.82-72.58%) and did not affect the *V*_*maxapp*_ with an increase of the substrate ACh concentration (Table 3).

**Table 3.** Effect of Pd(II) complex on *K*_Iapp_ and *V*_maxapp_ of *Bungarus sindanus* (krait) venom AChE. The *V*_maxapp_ and *K*_Iapp_ were determined from Dixon plot of Figure 4 for snake venom acetylcholinesterase. The *V*_maxapp_ is equal to the reciprocal of y-axis intersection of each line for each AcSCh/BuSCh concentration while *K*_Iapp_ is equal to the x-axis intersection in Dixon plot.

[**ASCh**] **(mM)**	K_Iapp_ (µM/mL)	% Increase	V_maxapp_(µmol/min per µM)
0.05	0.0912	0	32.2
0.1	0.064	30	32.2
0.25	0.0426	54	32.2
0.5	0.0292	68	31.25
1.0	0.025	73	30.3

### **Determination of inhibitory constants (*K***_i_ **)**

**Figure 6.**
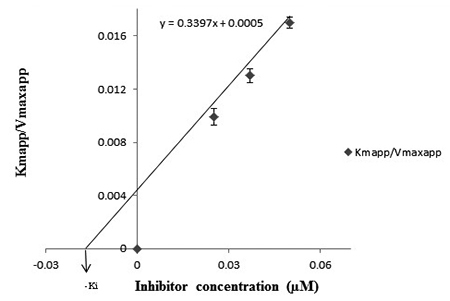
Determination of inhibitory constant (*Ki*).

*K*_i_ (inhibitory constant) was found to be 0.029 µM (Figure 6). The values are presented in Table 1.

## Discussion

Acetylcholinesterase (AChE) is a family of enzymes that catalyzes the hydrolysis of the neurotransmitter acetylcholine (ACh) into choline and acetic acid, a reaction necessary to allow a cholinergic neuron to return to its resting state after activation. AChE is a serine hydrolase mainly found at neuromuscular junctions and cholinergic brain synapses. Its principal biological role is termination of impulse transmission at cholinergic synapses by rapid hydrolysis of the neurotransmitter ACh to acetate and choline.

In the present study Pd(II) complex (Figure 1) inhibited cholinesterase in *Bungarus sindanus* venom. We observed that at fixed concentration 0.5 mM of substrate Pd(II) complex showed 61 % inhibition of acetylcholinesterase activity, (Figure 2). Also at a fixed concentration 0.5 mM of substrate (Figure 3) Pd (II) complex showed 50% inhibition at concentration of 0.043 µM/mL (Table 1). In the comparative study, the Pd(II) complex IC_50_ value is very close to AChE traditional inhibitors (tacrine, rivastigmine and galantamine) [45, 46].

In the present study competitive inhibition was observed with venom AChE for the kinetic analysis (Figure 4). In this case the *K*_*m*_ increased from 0.108 to 0.310 mM (45.74 to 318.35%) and the *V*_*max*_ was unchanged (Table 2). This behavior was indicated by the line weaver Burk double reciprocal plot [47]. Pd(II) complex inhibited AChE in*Bungarus sindanus*(krait) venom using ACh as a substrate. In such type of inhibition, the inhibitor competes with the substrate for binding to the active site of the enzyme, and thus; adequate substrate molecules can move the inhibitor from the enzyme active site. The results of the Lineweaver-Burk analysis indicate that Pd(II) complex inhibited snake venom AChE in a dose-dependent mode [47]. The Pd(II) complex compete with substrate ACh for binding at the active site of the enzyme and it does not react with the active site but its function is to occupy or prevent the binding of any other molecule of the substrate. *K*_*m*_ (Michaelis-Menten constant of AChE-ACh into AChE and product) and *K*_i_ (inhibitory constant) were estimated from (Figure 4 and 6) and were found to be 0.4 mM and 0.029 µM (Table 1). The *K*_*Iapp*_ and *V*_*maxapp*_ were observed for AChE (Figure 5) i.e. *K*_*Iapp*_ was found to increase from 0.0912 to 0.025 µM (29.82-72.58%) and did not affect the *V*_*maxapp*_ with increase of the substrate ACh concentration (Table 3). 

 In the present study, it is shown that Pd(II) complex act in a scorpion-like fashion by binding with the peripheral site (p-site) and blocking the entry of substrate ACh toward the active site of the AChE enzyme [48]. These results are also supported by the study of Harel [49] which confirmed that the crystal structure of antidepressant tacrine [an inhibitor of AChE enzyme with a structure similar to that of Pd(II) complex].

## Conclusion

Based on the kinetic parameter of AChE inhibition, we presume that due to the structural resemblance and ionic properties, Pd(II) complex compete with substrate ACh to bind with the active site of the enzyme. Furthermore, the results show that Pd(II) complex can be considered as an inhibitor of Krait snake venom AChE.
